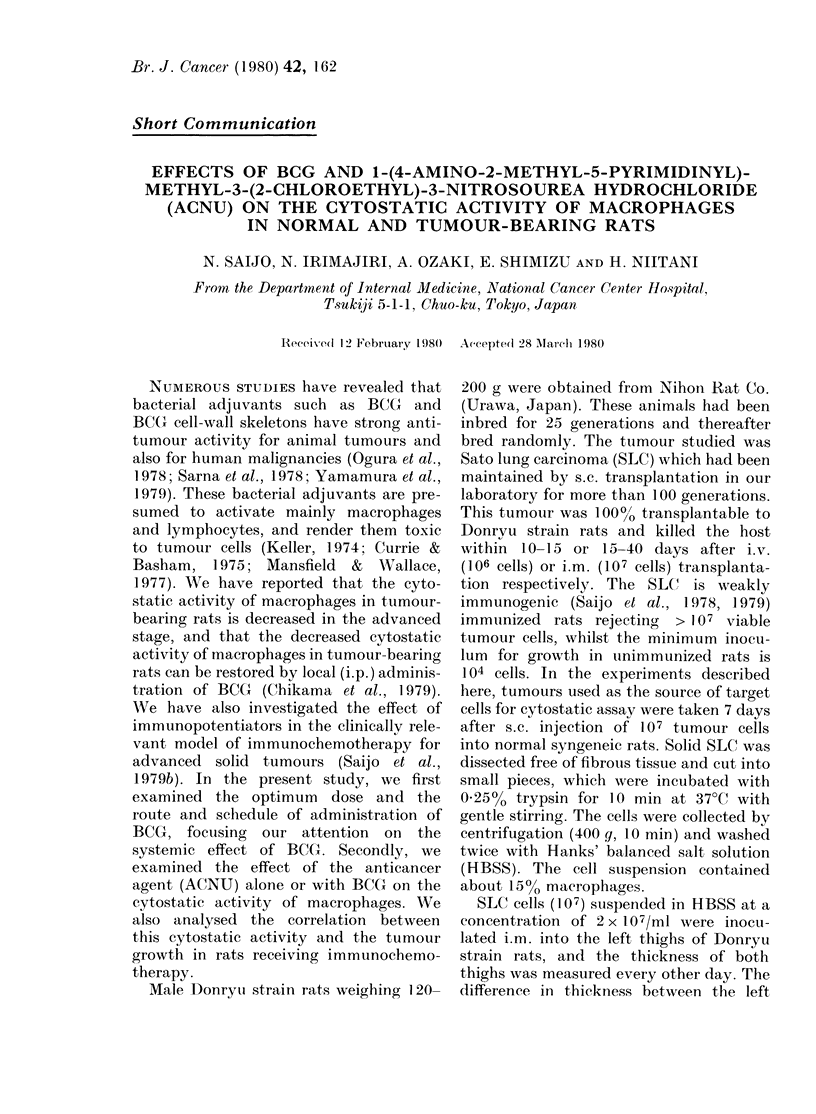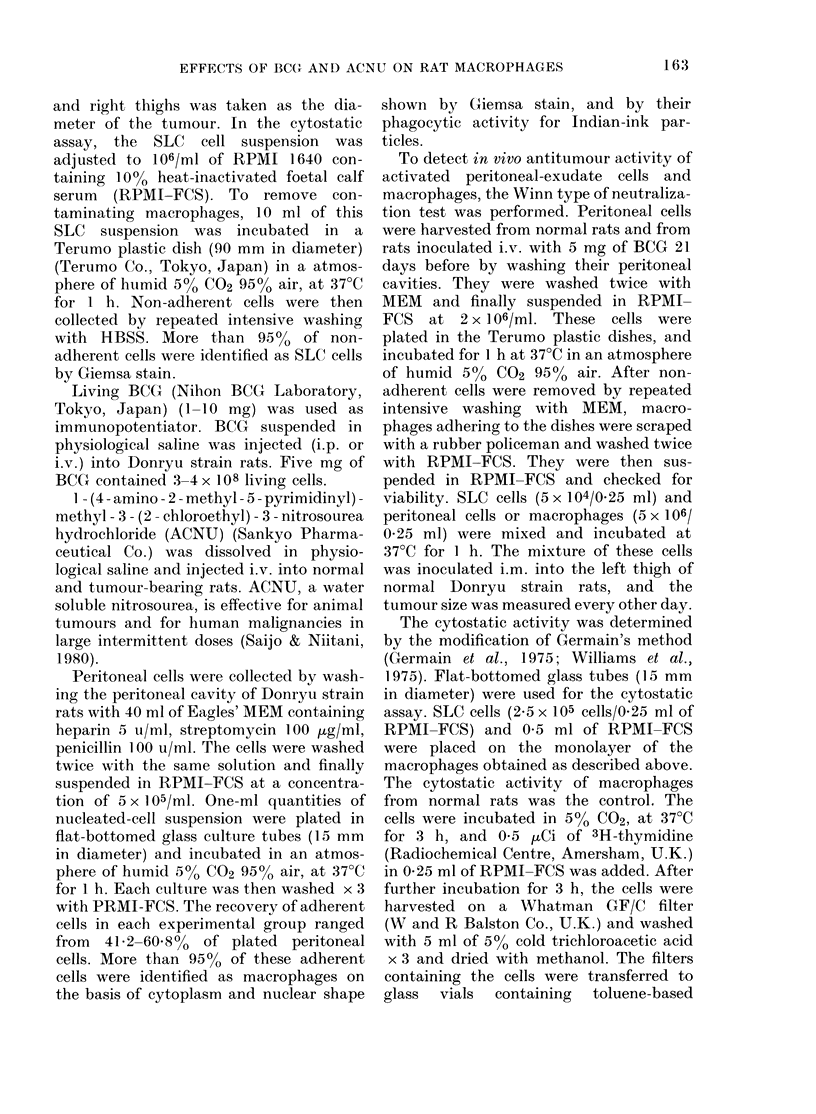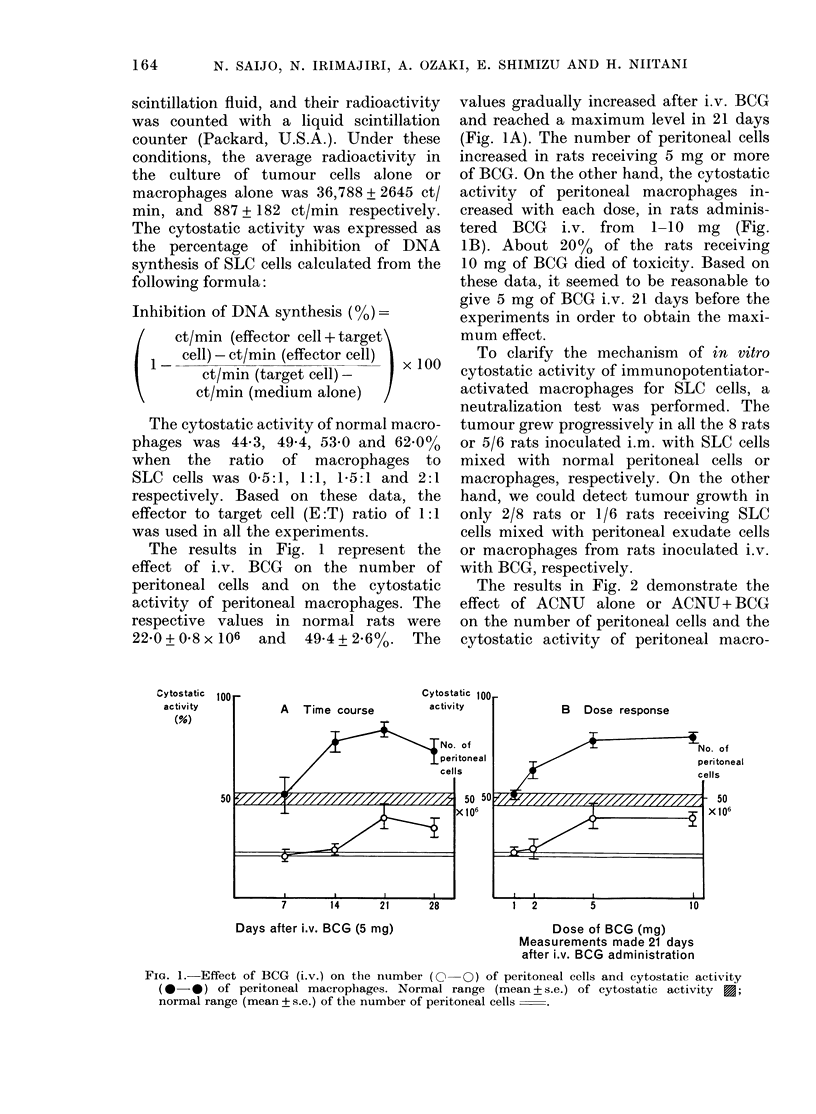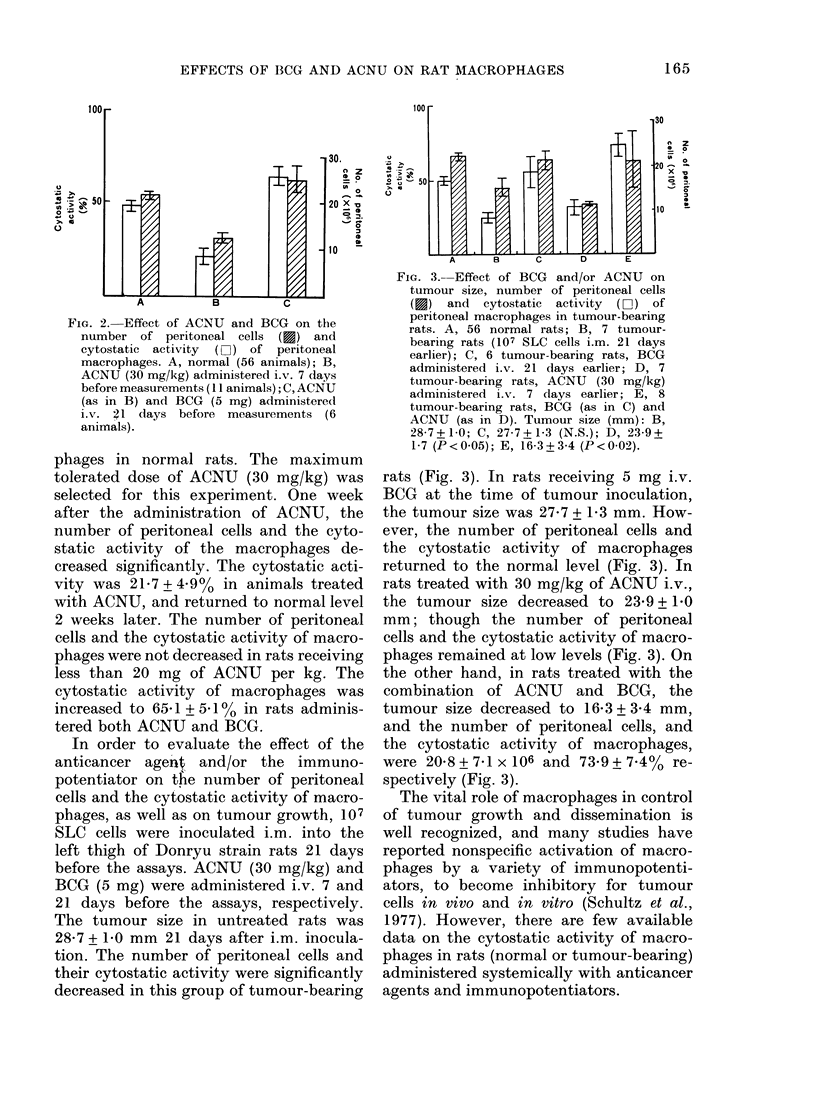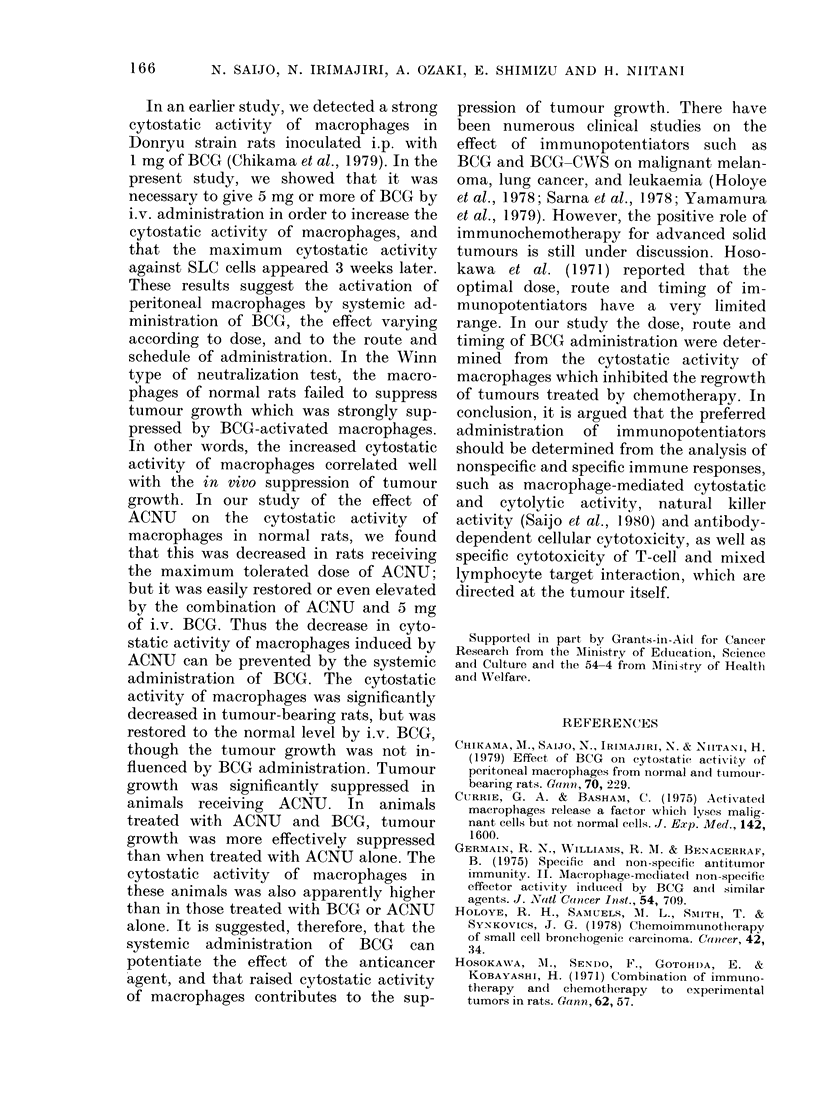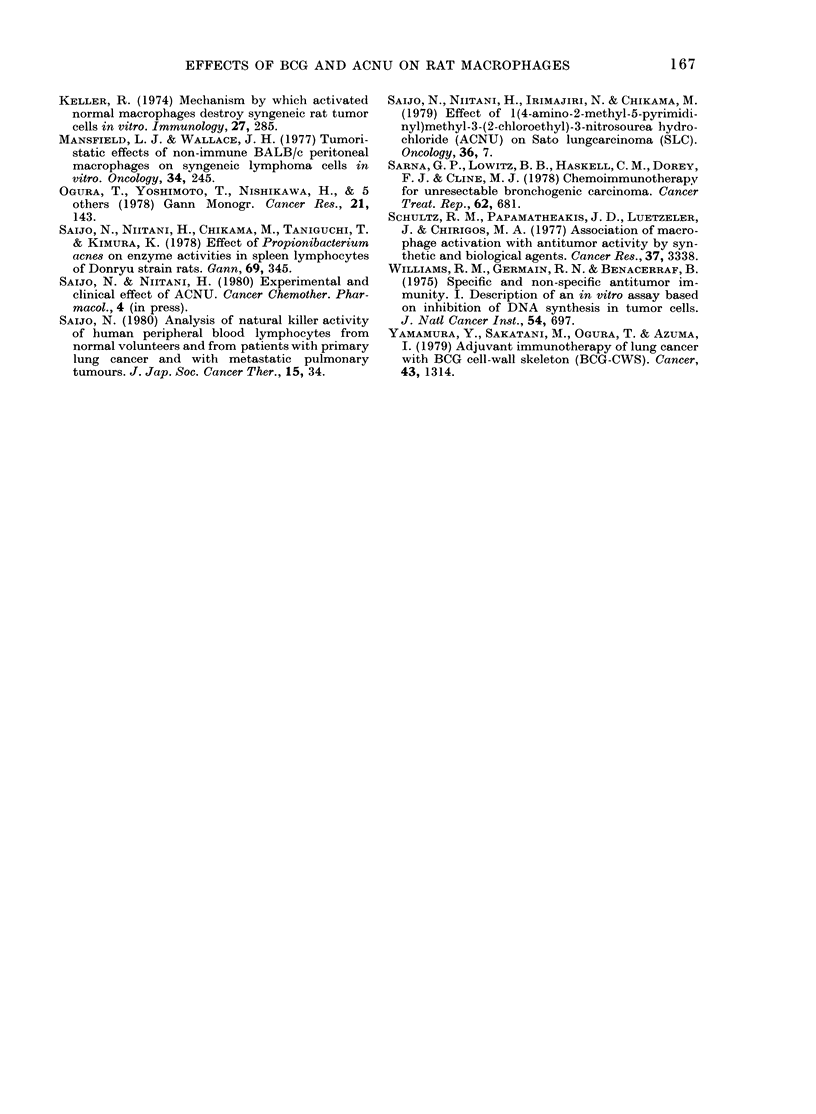# Effects of BCG and 1-(4-amino-2-methyl-5-pyrimidinyl)-methyl-3-(2-chloroethyl)-3-nitrosourea hydrochloride (ACNU) on the cytostatic activity of macrophages in normal and tumour-bearing rats.

**DOI:** 10.1038/bjc.1980.216

**Published:** 1980-07

**Authors:** N. Saijo, N. Irimajiri, A. Ozaki, E. Shimizu, H. Niitani


*Br. J. Cancer* (1980) **42**, 162

***Short Communication***

### EFFECTS OF BCG AND 1-(4-AMINO-2-METHYL-5-PYRIMIDINYL)-METHYL-3-(2-CHLOROETHYL)-3-NITROSOUREA HYDROCHLORIDE (ACNU) ON THE CYTOSTATIC ACTIVITY OF MACROPHAGES IN NORMAL AND TUMOUR-BEARING RATS

N. SAIJO, N. IRIMAJIRI, A. OZAKI, E. SHIMIZU AND H. NIITANI

*From the Department of Internal Medicine, National Cancer Center Hospital, Tsukiji* 5-1-1, *Chuo-ku, Tokyo, Japan*

Received 12 February 1980 Accepted 28 March 1980

NUMEROUS STUDIES have revealed that bacterial adjuvants such as BCG and BCG cell-wall skeletons have strong anti-tumour activity for animal tumours and also for human malignancies (Ogura *et al.*, 1978; Sarna *et al.*, 1978; Yamamura *et al.*, 1979). These bacterial adjuvants are presumed to activate mainly macrophages and lymphocytes, and render them toxic to tumour cells (Keller, 1974; Currie & Basham, 1975; Mansfield & Wallace, 1977). We have reported that the cytostatic activity of macrophages in tumour-bearing rats is decreased in the advanced stage, and that the decreased cytostatic activity of macrophages in tumour-bearing rats can be restored by local (i.p.) administration of BCG (Chikama *et al.*, 1979). We have also investigated the effect of immunopotentiators in the clinically relevant model of immunochemotherapy for advanced solid tumours (Saijo *et al.*, 1979*b*). In the present study, we first examined the optimum dose and the route and schedule of administration of BCG, focusing our attention on the systemic effect of BCG. Secondly, we examined the effect of the anticancer agent (ACNU) alone or with BCG on the cytostatic activity of macrophages. We also analysed the correlation between this cytostatic activity and the tumour growth in rats receiving immunochemotherapy.

Male Donryu strain rats weighing 120–200 g were obtained from Nihon Rat Co. (Urawa, Japan). These animals had been inbred for 25 generations and thereafter bred randomly. The tumour studied was Sato lung carcinoma (SLC) which had been maintained by s.c. transplantation in our laboratory for more than 100 generations. This tumour was 100% transplantable to Donryu strain rats and killed the host within 10–15 or 15–40 days after i.v. ($10^6$ cells) or i.m. ($10^7$ cells) transplantation respectively. The SLC is weakly immunogenic (Saijo *et al.*, 1978, 1979) immunized rats rejecting $>10^7$ viable tumour cells, whilst the minimum inoculum for growth in unimmunized rats is $10^4$ cells. In the experiments described here, tumours used as the source of target cells for cytostatic assay were taken 7 days after s.c. injection of $10^7$ tumour cells into normal syngeneic rats. Solid SLC was dissected free of fibrous tissue and cut into small pieces, which were incubated with 0·25% trypsin for 10 min at 37°C with gentle stirring. The cells were collected by centrifugation (400 *g*, 10 min) and washed twice with Hanks' balanced salt solution (HBSS). The cell suspension contained about 15% macrophages.

SLC cells ($10^7$) suspended in HBSS at a concentration of $2\times10^7$/ml were inoculated i.m. into the left thighs of Donryu strain rats, and the thickness of both thighs was measured every other day. The difference in thickness between the left and right thighs was taken as the diameter of the tumour. In the cytostatic assay, the SLC cell suspension was adjusted to $10^6$/ml of RPMI 1640 containing 10% heat-inactivated foetal calf serum (RPMI–FCS). To remove contaminating macrophages, 10 ml of this SLC suspension was incubated in a Terumo plastic dish (90 mm in diameter) (Terumo Co., Tokyo, Japan) in a atmosphere of humid 5% $CO_2$ 95% air, at 37°C for 1 h. Non-adherent cells were then collected by repeated intensive washing with HBSS. More than 95% of non-adherent cells were identified as SLC cells by Giemsa stain.

Living BCG (Nihon BCG Laboratory, Tokyo, Japan) (1–10 mg) was used as immunopotentiator. BCG suspended in physiological saline was injected (i.p. or i.v.) into Donryu strain rats. Five mg of BCG contained $3–4 \times 10^8$ living cells.

1-(4-amino-2-methyl-5-pyrimidinyl)-methyl-3-(2-chloroethyl)-3-nitrosourea hydrochloride (ACNU) (Sankyo Pharmaceutical Co.) was dissolved in physiological saline and injected i.v. into normal and tumour-bearing rats. ACNU, a water soluble nitrosourea, is effective for animal tumours and for human malignancies in large intermittent doses (Saijo & Niitani, 1980).

Peritoneal cells were collected by washing the peritoneal cavity of Donryu strain rats with 40 ml of Eagles' MEM containing heparin 5 u/ml, streptomycin 100 $\mu$g/ml, penicillin 100 u/ml. The cells were washed twice with the same solution and finally suspended in RPMI–FCS at a concentration of $5 \times 10^5$/ml. One-ml quantities of nucleated-cell suspension were plated in flat-bottomed glass culture tubes (15 mm in diameter) and incubated in an atmosphere of humid 5% $CO_2$ 95% air, at 37°C for 1 h. Each culture was then washed $\times 3$ with PRMI-FCS. The recovery of adherent cells in each experimental group ranged from 41·2–60·8% of plated peritoneal cells. More than 95% of these adherent cells were identified as macrophages on the basis of cytoplasm and nuclear shape shown by Giemsa stain, and by their phagocytic activity for Indian-ink particles.

To detect *in vivo* antitumour activity of activated peritoneal-exudate cells and macrophages, the Winn type of neutralization test was performed. Peritoneal cells were harvested from normal rats and from rats inoculated i.v. with 5 mg of BCG 21 days before by washing their peritoneal cavities. They were washed twice with MEM and finally suspended in RPMI–FCS at $2 \times 10^6$/ml. These cells were plated in the Terumo plastic dishes, and incubated for 1 h at 37°C in an atmosphere of humid 5% $CO_2$ 95% air. After non-adherent cells were removed by repeated intensive washing with MEM, macrophages adhering to the dishes were scraped with a rubber policeman and washed twice with RPMI–FCS. They were then suspended in RPMI–FCS and checked for viability. SLC cells ($5 \times 10^4$/0·25 ml) and peritoneal cells or macrophages ($5 \times 10^6$/0·25 ml) were mixed and incubated at 37°C for 1 h. The mixture of these cells was inoculated i.m. into the left thigh of normal Donryu strain rats, and the tumour size was measured every other day.

The cytostatic activity was determined by the modification of Germain's method (Germain *et al.*, 1975; Williams *et al.*, 1975). Flat-bottomed glass tubes (15 mm in diameter) were used for the cytostatic assay. SLC cells ($2·5 \times 10^5$ cells/0·25 ml of RPMI–FCS) and 0·5 ml of RPMI–FCS were placed on the monolayer of the macrophages obtained as described above. The cytostatic activity of macrophages from normal rats was the control. The cells were incubated in 5% $CO_2$, at 37°C for 3 h, and 0·5 $\mu$Ci of $^3$H-thymidine (Radiochemical Centre, Amersham, U.K.) in 0·25 ml of RPMI–FCS was added. After further incubation for 3 h, the cells were harvested on a Whatman GF/C filter (W and R Balston Co., U.K.) and washed with 5 ml of 5% cold trichloroacetic acid $\times 3$ and dried with methanol. The filters containing the cells were transferred to glass vials containing toluene-based scintillation fluid, and their radioactivity was counted with a liquid scintillation counter (Packard, U.S.A.). Under these conditions, the average radioactivity in the culture of tumour cells alone or macrophages alone was $36{,}788 \pm 2645$ ct/min, and $887 \pm 182$ ct/min respectively. The cytostatic activity was expressed as the percentage of inhibition of DNA synthesis of SLC cells calculated from the following formula:

$$\text{Inhibition of DNA synthesis (\%)} = \left(1 - \frac{\text{ct/min (effector cell + target cell)} - \text{ct/min (effector cell)}}{\text{ct/min (target cell)} - \text{ct/min (medium alone)}}\right) \times 100$$

The cytostatic activity of normal macrophages was 44·3, 49·4, 53·0 and 62·0% when the ratio of macrophages to SLC cells was 0·5:1, 1:1, 1·5:1 and 2:1 respectively. Based on these data, the effector to target cell (E:T) ratio of 1:1 was used in all the experiments.

The results in Fig. 1 represent the effect of i.v. BCG on the number of peritoneal cells and on the cytostatic activity of peritoneal macrophages. The respective values in normal rats were $22{\cdot}0 \pm 0{\cdot}8 \times 10^6$ and $49{\cdot}4 \pm 2{\cdot}6\%$. The values gradually increased after i.v. BCG and reached a maximum level in 21 days (Fig. 1A). The number of peritoneal cells increased in rats receiving 5 mg or more of BCG. On the other hand, the cytostatic activity of peritoneal macrophages increased with each dose, in rats administered BCG i.v. from 1–10 mg (Fig. 1B). About 20% of the rats receiving 10 mg of BCG died of toxicity. Based on these data, it seemed to be reasonable to give 5 mg of BCG i.v. 21 days before the experiments in order to obtain the maximum effect.

To clarify the mechanism of *in vitro* cytostatic activity of immunopotentiator-activated macrophages for SLC cells, a neutralization test was performed. The tumour grew progressively in all the 8 rats or 5/6 rats inoculated i.m. with SLC cells mixed with normal peritoneal cells or macrophages, respectively. On the other hand, we could detect tumour growth in only 2/8 rats or 1/6 rats receiving SLC cells mixed with peritoneal exudate cells or macrophages from rats inoculated i.v. with BCG, respectively.

The results in Fig. 2 demonstrate the effect of ACNU alone or ACNU+BCG on the number of peritoneal cells and the cytostatic activity of peritoneal macro-

FIG. 1.—Effect of BCG (i.v.) on the number (○—○) of peritoneal cells and cytostatic activity (●—●) of peritoneal macrophages. Normal range (mean ± s.e.) of cytostatic activity ▨; normal range (mean ± s.e.) of the number of peritoneal cells ═══.

FIG. 2.—Effect of ACNU and BCG on the number of peritoneal cells (▨) and cytostatic activity (□) of peritoneal macrophages. A, normal (56 animals); B, ACNU (30 mg/kg) administered i.v. 7 days before measurements (11 animals); C, ACNU (as in B) and BCG (5 mg) administered i.v. 21 days before measurements (6 animals).

FIG. 3.—Effect of BCG and/or ACNU on tumour size, number of peritoneal cells (▨) and cytostatic activity (□) of peritoneal macrophages in tumour-bearing rats. A, 56 normal rats; B, 7 tumour-bearing rats ($10^7$ SLC cells i.m. 21 days earlier); C, 6 tumour-bearing rats, BCG administered i.v. 21 days earlier; D, 7 tumour-bearing rats, ACNU (30 mg/kg) administered i.v. 7 days earlier; E, 8 tumour-bearing rats, BCG (as in C) and ACNU (as in D). Tumour size (mm): B, $28{\cdot}7 \pm 1{\cdot}0$; C, $27{\cdot}7 \pm 1{\cdot}3$ (N.S.); D, $23{\cdot}9 \pm 1{\cdot}7$ ($P < 0{\cdot}05$); E, $16{\cdot}3 \pm 3{\cdot}4$ ($P < 0{\cdot}02$).

phages in normal rats. The maximum tolerated dose of ACNU (30 mg/kg) was selected for this experiment. One week after the administration of ACNU, the number of peritoneal cells and the cytostatic activity of the macrophages decreased significantly. The cytostatic activity was $21{\cdot}7 \pm 4{\cdot}9\%$ in animals treated with ACNU, and returned to normal level 2 weeks later. The number of peritoneal cells and the cytostatic activity of macrophages were not decreased in rats receiving less than 20 mg of ACNU per kg. The cytostatic activity of macrophages was increased to $65{\cdot}1 \pm 5{\cdot}1\%$ in rats administered both ACNU and BCG.

In order to evaluate the effect of the anticancer agent and/or the immunopotentiator on the number of peritoneal cells and the cytostatic activity of macrophages, as well as on tumour growth, $10^7$ SLC cells were inoculated i.m. into the left thigh of Donryu strain rats 21 days before the assays. ACNU (30 mg/kg) and BCG (5 mg) were administered i.v. 7 and 21 days before the assays, respectively. The tumour size in untreated rats was $28{\cdot}7 \pm 1{\cdot}0$ mm 21 days after i.m. inoculation. The number of peritoneal cells and their cytostatic activity were significantly decreased in this group of tumour-bearing rats (Fig. 3). In rats receiving 5 mg i.v. BCG at the time of tumour inoculation, the tumour size was $27{\cdot}7 \pm 1{\cdot}3$ mm. However, the number of peritoneal cells and the cytostatic activity of macrophages returned to the normal level (Fig. 3). In rats treated with 30 mg/kg of ACNU i.v., the tumour size decreased to $23{\cdot}9 \pm 1{\cdot}0$ mm; though the number of peritoneal cells and the cytostatic activity of macrophages remained at low levels (Fig. 3). On the other hand, in rats treated with the combination of ACNU and BCG, the tumour size decreased to $16{\cdot}3 \pm 3{\cdot}4$ mm, and the number of peritoneal cells, and the cytostatic activity of macrophages, were $20{\cdot}8 \pm 7{\cdot}1 \times 10^6$ and $73{\cdot}9 \pm 7{\cdot}4\%$ respectively (Fig. 3).

The vital role of macrophages in control of tumour growth and dissemination is well recognized, and many studies have reported nonspecific activation of macrophages by a variety of immunopotentiators, to become inhibitory for tumour cells *in vivo* and *in vitro* (Schultz *et al.*, 1977). However, there are few available data on the cytostatic activity of macrophages in rats (normal or tumour-bearing) administered systemically with anticancer agents and immunopotentiators.

In an earlier study, we detected a strong cytostatic activity of macrophages in Donryu strain rats inoculated i.p. with 1 mg of BCG (Chikama *et al.*, 1979). In the present study, we showed that it was necessary to give 5 mg or more of BCG by i.v. administration in order to increase the cytostatic activity of macrophages, and that the maximum cytostatic activity against SLC cells appeared 3 weeks later. These results suggest the activation of peritoneal macrophages by systemic administration of BCG, the effect varying according to dose, and to the route and schedule of administration. In the Winn type of neutralization test, the macrophages of normal rats failed to suppress tumour growth which was strongly suppressed by BCG-activated macrophages. In other words, the increased cytostatic activity of macrophages correlated well with the *in vivo* suppression of tumour growth. In our study of the effect of ACNU on the cytostatic activity of macrophages in normal rats, we found that this was decreased in rats receiving the maximum tolerated dose of ACNU; but it was easily restored or even elevated by the combination of ACNU and 5 mg of i.v. BCG. Thus the decrease in cytostatic activity of macrophages induced by ACNU can be prevented by the systemic administration of BCG. The cytostatic activity of macrophages was significantly decreased in tumour-bearing rats, but was restored to the normal level by i.v. BCG, though the tumour growth was not influenced by BCG administration. Tumour growth was significantly suppressed in animals receiving ACNU. In animals treated with ACNU and BCG, tumour growth was more effectively suppressed than when treated with ACNU alone. The cytostatic activity of macrophages in these animals was also apparently higher than in those treated with BCG or ACNU alone. It is suggested, therefore, that the systemic administration of BCG can potentiate the effect of the anticancer agent, and that raised cytostatic activity of macrophages contributes to the suppression of tumour growth. There have been numerous clinical studies on the effect of immunopotentiators such as BCG and BCG–CWS on malignant melanoma, lung cancer, and leukaemia (Holoye *et al.*, 1978; Sarna *et al.*, 1978; Yamamura *et al.*, 1979). However, the positive role of immunochemotherapy for advanced solid tumours is still under discussion. Hosokawa *et al.* (1971) reported that the optimal dose, route and timing of immunopotentiators have a very limited range. In our study the dose, route and timing of BCG administration were determined from the cytostatic activity of macrophages which inhibited the regrowth of tumours treated by chemotherapy. In conclusion, it is argued that the preferred administration of immunopotentiators should be determined from the analysis of nonspecific and specific immune responses, such as macrophage-mediated cytostatic and cytolytic activity, natural killer activity (Saijo *et al.*, 1980) and antibody-dependent cellular cytotoxicity, as well as specific cytotoxicity of T-cell and mixed lymphocyte target interaction, which are directed at the tumour itself.

Supported in part by Grants-in-Aid for Cancer Research from the Ministry of Education, Science and Culture and the 54–4 from Ministry of Health and Welfare.

### REFERENCES

Chikama, M., Saijo, N., Irimajiri, N. & Niitani, H. (1979) Effect of BCG on cytostatic activity of peritoneal macrophages from normal and tumour-bearing rats. *Gann*, **70**, 229.

Currie, G. A. & Basham, C. (1975) Activated macrophages release a factor which lyses malignant cells but not normal cells. *J. Exp. Med.*, **142**, 1600.

Germain, R. N., Williams, R. M. & Benacerraf, B. (1975) Specific and non-specific antitumor immunity. II. Macrophage-mediated non-specific effector activity induced by BCG and similar agents. *J. Natl Cancer Inst.*, **54**, 709.

Holoye, R. H., Samuels, M. L., Smith, T. & Synkovics, J. G. (1978) Chemoimmunotherapy of small cell bronchogenic carcinoma. *Cancer*, **42**, 34.

Hosokawa, M., Sendo, F., Gotohda, E. & Kobayashi, H. (1971) Combination of immunotherapy and chemotherapy to experimental tumors in rats. *Gann*, **62**, 57.

KELLER, R. (1974) Mechanism by which activated normal macrophages destroy syngeneic rat tumor cells *in vitro*. *Immunology*, **27**, 285.

MANSFIELD, L. J. & WALLACE, J. H. (1977) Tumoristatic effects of non-immune BALB/c peritoneal macrophages on syngeneic lymphoma cells *in vitro*. *Oncology*, **34**, 245.

OGURA, T., YOSHIMOTO, T., NISHIKAWA, H., & 5 others (1978) Gann Monogr. *Cancer Res.*, **21**, 143.

SAIJO, N., NIITANI, H., CHIKAMA, M., TANIGUCHI, T. & KIMURA, K. (1978) Effect of *Propionibacterium acnes* on enzyme activities in spleen lymphocytes of Donryu strain rats. *Gann*, **69**, 345.

SAIJO, N. & NIITANI, H. (1980) Experimental and clinical effect of ACNU. *Cancer Chemother. Pharmacol.*, **4** (in press).

SAIJO, N. (1980) Analysis of natural killer activity of human peripheral blood lymphocytes from normal volunteers and from patients with primary lung cancer and with metastatic pulmonary tumours. *J. Jap. Soc. Cancer Ther.*, **15**, 34.

SAIJO, N., NIITANI, H., IRIMAJIRI, N. & CHIKAMA, M. (1979) Effect of 1(4-amino-2-methyl-5-pyrimidinyl)methyl-3-(2-chloroethyl)-3-nitrosourea hydrochloride (ACNU) on Sato lungcarcinoma (SLC). *Oncology*, **36**, 7.

SARNA, G. P., LOWITZ, B. B., HASKELL, C. M., DOREY, F. J. & CLINE, M. J. (1978) Chemoimmunotherapy for unresectable bronchogenic carcinoma. *Cancer Treat. Rep.*, **62**, 681.

SCHULTZ, R. M., PAPAMATHEAKIS, J. D., LUETZELER, J. & CHIRIGOS, M. A. (1977) Association of macrophage activation with antitumor activity by synthetic and biological agents. *Cancer Res.*, **37**, 3338.

WILLIAMS, R. M., GERMAIN, R. N. & BENACERRAF, B. (1975) Specific and non-specific antitumor immunity. I. Description of an *in vitro* assay based on inhibition of DNA synthesis in tumor cells. *J. Natl Cancer Inst.*, **54**, 697.

YAMAMURA, Y., SAKATANI, M., OGURA, T. & AZUMA, I. (1979) Adjuvant immunotherapy of lung cancer with BCG cell-wall skeleton (BCG-CWS). *Cancer*, **43**, 1314.